# Substance deposition assessment in obstructed pulmonary system through numerical characterization of airflow and inhaled particles attributes

**DOI:** 10.1186/s12911-017-0561-y

**Published:** 2017-12-20

**Authors:** Antonios Lalas, Stavros Nousias, Dimitrios Kikidis, Aris Lalos, Gerasimos Arvanitis, Christos Sougles, Konstantinos Moustakas, Konstantinos Votis, Sylvia Verbanck, Omar Usmani, Dimitrios Tzovaras

**Affiliations:** 1 Information Technologies Institute, Centre for Research and Technology - Hellas (CERTH), Thessaloniki, Greece; 2 Department of Electrical and Computer Engineering, University of Patras, Patra, Greece; 3 Respiratory Division, University Hospital UZ Brussel, Vrije Universiteit Brussel, Brussels, Belgium; 4 National Heart and Lung Institute (NHLI), Imperial College London and Royal Brompton Hospital, London, UK

**Keywords:** Computational fluid dynamics, Fluid particle tracing, Obstructive lung diseases, Aerosol deposition, Human airways

## Abstract

**Background:**

Chronic obstructive pulmonary disease (COPD) and asthma are considered as the two most widespread obstructive lung diseases, whereas they affect more than 500 million people worldwide. Unfortunately, the requirement for detailed geometric models of the lungs in combination with the increased computational resources needed for the simulation of the breathing did not allow great progress to be made in the past for the better understanding of inflammatory diseases of the airways through detailed modelling approaches. In this context, computational fluid dynamics (CFD) simulations accompanied by fluid particle tracing (FPT) analysis of the inhaled ambient particles are deemed critical for lung function assessment. Also they enable the understanding of particle depositions on the airways of patients, since these accumulations may affect or lead to inflammations. In this direction, the current study conducts an initial investigation for the better comprehension of particle deposition within the lungs. More specifically, accurate models of the airways obstructions that relate to pulmonary disease are developed and a thorough assessment of the airflow behavior together with identification of the effects of inhaled particle properties, such as size and density, is conducted. Our approach presents a first step towards an effective personalization of pulmonary treatment in regards to the geometric characteristics of the lungs and the in depth understanding of airflows within the airways.

**Methods:**

A geometry processing technique involving contraction algorithms is established and used to employ the different respiratory arrangements associated with lung related diseases that exhibit airways obstructions. Apart from the normal lung case, two categories of obstructed cases are examined, i.e. models with obstructions in both lungs and models with narrowings in the right lung only. Precise assumptions regarding airflow and deposition fraction (DF) over various sections of the lungs are drawn by simulating these distinct incidents through the finite volume method (FVM) and particularly the CFD and FPT algorithms. Moreover, a detailed parametric analysis clarifies the effects of the particles size and density in terms of regional deposition upon several parts of the pulmonary system. In this manner, the deposition pattern of various substances can be assessed.

**Results:**

For the specific case of the unobstructed lung model most particles are detected on the right lung (48.56% of total, when the air flowrate is 12.6 L/min), a fact that is also true when obstructions arise symmetrically in both lungs (51.45% of total, when the air flowrate is 6.06 L/min and obstructions occur after the second generation). In contrast, when narrowings are developed on the right lung only, most particles are pushed on the left section (68.22% of total, when the air flowrate is 11.2 L/min) indicating that inhaled medication is generally deposited away from the areas of inflammation. This observation is useful when designing medical treatment of lung diseases. Furthermore, particles with diameters from 1 μm to 10 μm are shown to be mainly deposited on the lower airways, whereas particles with diameters of 20 μm and 30 μm are mostly accumulated in the upper airways. As a result, the current analysis indicates increased DF levels in the upper airways when the particle diameter is enlarged. Additionally, when the particles density increases from 1000 Kg/m^3^ to 2000 Kg/m^3^, the DF is enhanced on every generation and for all cases investigated herein. The results obtained by our simulations provide an accurate and quantitative estimation of all important parameters involved in lung modeling.

**Conclusions:**

The treatment of respiratory diseases with inhaled medical substances can be advanced by the clinical use of accurate CFD and FPT simulations and specifically by evaluating the deposition of inhaled particles in a regional oriented perspective in regards to different particle sizes and particle densities. Since a drug with specific characteristics (i.e. particle size and density) exhibits maximum deposition on particular lung areas, the current study provides initial indications to a qualified physician for proper selection of medication.

## Background

Chronic obstructive pulmonary disease (COPD) and asthma are deemed as the two most frequent life-long inflammatory maladies of the respiratory system affecting over 500 million people worldwide. Since the assessment of their severity is of paramount importance, several clinical tools established by ICT infrastructure [1, 2] have been utilized for this purpose. In addition, identification of markers and factors that induce asthma attacks is considered critical to enable instant and accurate treatment. A significant indicator of asthma disease occurrence is attributed to the ventilation heterogeneity due to small airways dysfunction [3]. Also, asthma exacerbations in asthmatic children are stimulated by airborne fine water particles, which are denoted as important etiological factors [4]. In this framework, the basic methodology when implementing severity estimation tools is formulated by computational fluid dynamics (CFD) simulations [5, 6] in conjunction with fluid particle tracing (FPT) analysis of the inhaled ambient particles [7–9]. In this manner, the critical features of airflow, in accordance with the effect of particle density and size on particle deposition in the respiratory airways can be studied. As of today, the inspiration and expiration procedures of lung function have been analyzed by employing several CFD models [10–12]. In addition, various approaches of lung modeling present calculations of the particles characteristics [13–16] by taking into account the data gathered by the CFD analysis.

Moreover, a method that utilizes multidetector computer tomography (MDCT) volumetric data sets, obtained at diverse inflation levels, in order to assemble a multiscale breathing lung model has been introduced in [17]. Additional aspects of lung modeling have been introduced by [18] that relate to the modeling of respiratory system topology (conducting airways), lung function (regional ventilation and boundary conditions), as well as the features of airflow (particle transport based on the modeling of turbulent flow). Τhe pathophysiological mechanisms in COPD and asthma have been examined by introducing multi-scale computational models of the airways [19]. A structurally-inspired model of the lung acinus has been established [20, 21], which involves a bifurcating tree of alveolated airways with moving walls. Furthermore, the features of the airflow in the human respiratory system, between the 8th and 14th generations of the lung, have been studied [22]. The aforementioned approaches are capable of assessing the behavior of the airflow, as well as the inhaled particles, by employing numerous turbulence models. However, the airflow characteristics have not been clarified at the upper generations, i.e. between the 1st and the 5th, especially when narrowings are involved.

The dose evaluation of ambient particles transported from a subway microenvironment into the human respiratory tract, as well as the significance of individual exposure to them, have been described by [23], so that to provide knowledge for the protection of public health. As an example, the accumulation of inhaled particles within the generations of the respiratory system of an adult woman for several circumstances, such as sleeping, sitting, light and heavy exercise breathing conditions, has been determined by a stochastic lung deposition model [24]. A stochastic lung model to investigate maximization of the amount of accumulated particles during a severe asthma attack [25] has been utilized as the most illustrative methodology to cope with the prediction of asthma exacerbations. Other approaches allow the assessment of the efficiency of the inhaler procedures embraced for treating asthma [26], along with the pharmaceutical components in the form of inhaled particles [27, 28]. Experimental investigation of inhaled aerosol distribution in human pulmonary system based on a 3D printed lung model has been presented by [29], along with validation of the associated CFD simulations. The lack of personalization in estimating the necessary dosage of inhaled medicines is considered an important shortcoming of the existing technologies for asthma treatment. An initial investigation regarding this estimation has been conducted in [30], taking into account the airflow characteristics at the upper generations.

The objective of this work is to develop precise models of the airways narrowings that correlate to respiratory maladies. Moreover, we provide an extensive assessment of the airflow features together with clarification of the inhaled particle attributes as a next step towards attaining effective personalized treatment with properly tailored substances. To pursue these goals, a geometry processing methodology is established and employed for the formation of various important models of the pulmonary system. The proposed sequence mitigates several effects that degrade the quality of the surface models, thus facilitating the required simulations. Apart from the normal lung case, two obstructed cases are examined, i.e. a model with obstructions in both lungs and a model with obstructions in the right lung only. The partially obstructed model is evaluated only for the right lung to assess the effects of obstructions in the asymmetric behavior of the normal model, since most particles are deposited in the right section as shown by our initial investigations. These distinct cases are examined through the finite volume method (FVM) and specifically the CFD and FPT algorithms which allow accurate deductions about airflow and deposition fraction (DF) upon numerous parts of the lungs. In addition, a thorough parametric analysis is conducted to identify the effects of particle size and density on their deposition efficacy upon various sections of the pulmonary system. In this way, the behavior of various substances with diverse dosages can be assessed in terms of the deposition pattern that they exhibit. The data obtained demonstrate potential employment in personalized medical remedy of lung diseases by providing regional-oriented deposition information.

## Methods

### Structural modeling, parametrization and deformation of the lung

**Fig. 1:**
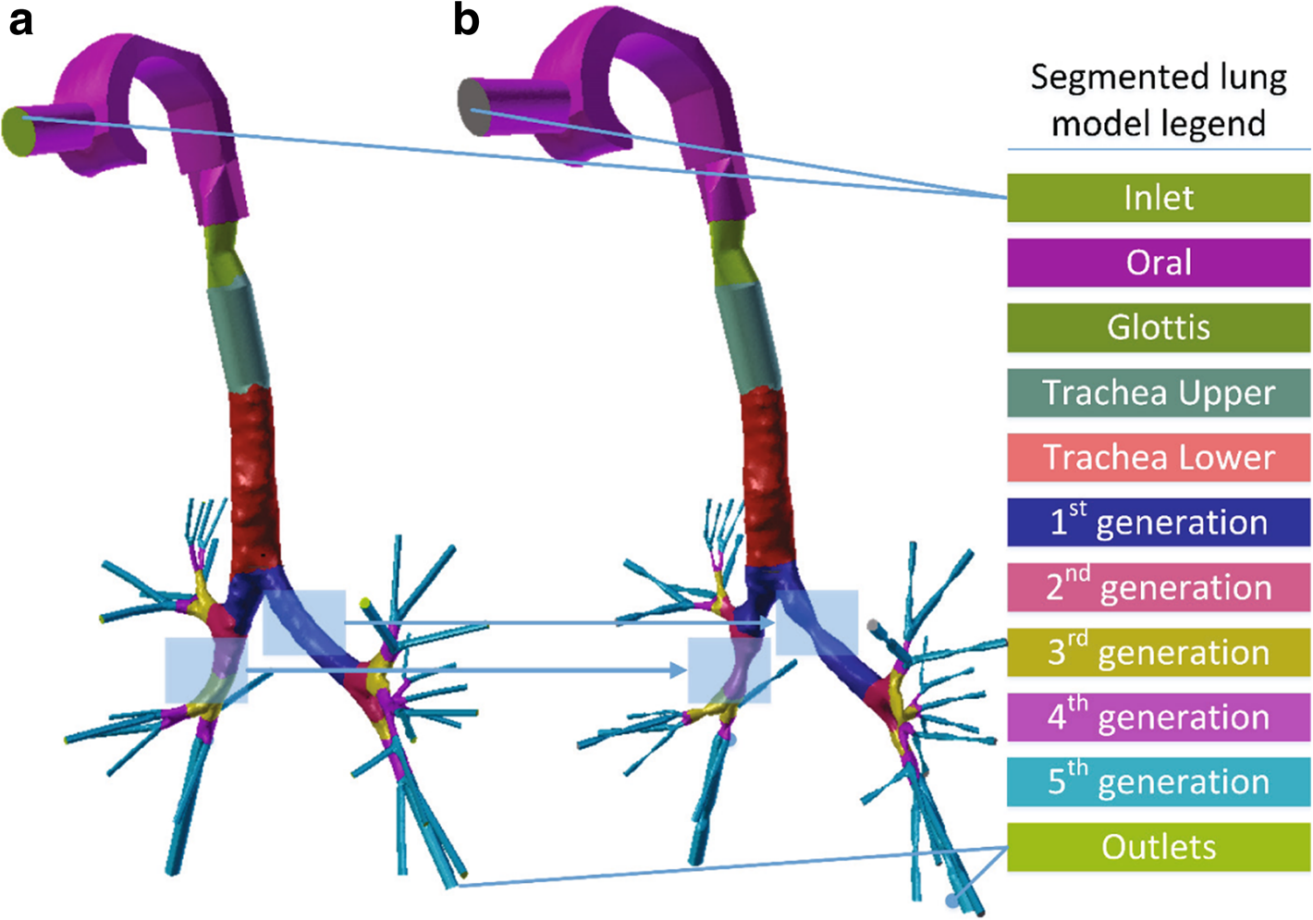
Patient personalized model of lung geometry up to the 5th generation of the pulmonary airways: (**a**) Normal case and (**b**) narrowed airways geometry (both lungs are obstructed)

Geometry processing sequences are established to allow the simulation of the effects of obstructions in the 3D lung topology. This approach enables the investigation of the impact of any narrowings on the airflow into the lung, by employing a CFD simulation. The proposed sequences are implemented directly on an already available 3D model of the lung [29], obtained from CT scans, by utilising state-of-the-art techniques. A Laplacian mesh contraction procedure in the direction of the inward normal is executed to iteratively apply contractions to the surface of a certain segment of the mesh. Several correction procedures are applied to mitigate degradation effects and abnormalities. Furthermore, the lung geometry is splitted into various parts, such as the generations and the outlets, as depicted in Fig. 1, to facilitate the analysis.

Our geometry approach is focused on polygon models whose surface is represented using triangles. Each triangle mesh $\mathcal{M}$ with *n* vertices can be denoted by two distinct sets $\mathcal{M}=\left(V,F\right)$ corresponding to the vertices (*V*) that represent the geometry information and the indexed faces (*F*) of the mesh. Each vertex can be represented as a point **v**_*i*_ = (*x*_*i*_, *y*_i_, *z*_*i*_) ∀  *i* = 1, …*n* and each centroid of a face as ${\mathbf{m}}_i=\frac{{\mathbf{v}}_{i1}+{\mathbf{v}}_{i2}+{\mathbf{v}}_{i3}}{3}\forall \kern0.5em i=1,\dots l$. A set of edges (*E*) can be directly derived from *V* and *F*, which correspond to the connectivity information. If we denote with **v** the vertices of a mesh, then each face can be represented by its centroid point **m**_*i*_, and its outward unit normal:

1 $$ {\hat{\mathbf{n}}}_{m_i}=\frac{\left({\mathbf{v}}_{i_2}-{\mathbf{v}}_{i_1}\right)\times \left({\mathbf{v}}_{i_3}-{\mathbf{v}}_{i_1}\right)}{\parallel \left({\mathbf{v}}_{i_2}-{\mathbf{v}}_{i_1}\right)\times \left({\mathbf{v}}_{i_3}-{\mathbf{v}}_{i_1}\right)\parallel}\forall i=1,\dots {n}_f $$

where ${\mathbf{v}}_{i_1}$, ${\mathbf{v}}_{i_2}$, ${\mathbf{v}}_{i_3}$ are the vertices that are related with face *f*_*i*_ and ${\widehat{\mathrm{n}}}_{\mathbf{m}}=\left[{\widehat{\mathbf{n}}}_{m_1}^T\kern0.5em {\widehat{\mathbf{n}}}_{m_2}^T\cdots \kern0.5em {\widehat{\mathbf{n}}}_{nf}^T\right]\in {\mathfrak{R}}^{3{n}_f\times 1}$.

In this section we use the following notation: Lower- and upper-case boldface letters are used to represent column vectors and matrices, respectively; calligraphic letters are used to denote sets. The entry in the *i*-th row and *j*-th column of a matrix **A** is represented by **A**_(*i*, *j*)_, while the *i*-th row and *j*-th column are denoted by **A**_(*i*, :)_ and **A**_(:, *j*)_, respectively. Also, (·)^*T*^ denotes transposition.

#### Structural modeling

Structural modelling of the lung must include all the features that describe the geometry of the lung either in detailed (e.g., 3D mesh) or in consice manner (e.g., 1D representation). Specifically, the 3D model is obtained from CT scans by employing state-of-the-art techniques and it consists of the set of vertices *V*, the set of edges *E* and the faces *F*. The 1D representation of the lung is extracted by applying skeletonization [31] approaches directly to the 3D model. In particular, the 3D mesh is iteratively contracted (simplified) to a zero-volume mesh until each node to be connected to three or only one nodes. The subsequent 1D representation consists also of a set of vertices *V* and edges *E*.

#### Lung geometry contraction in the direction of the inward normals

**Fig. 2:**
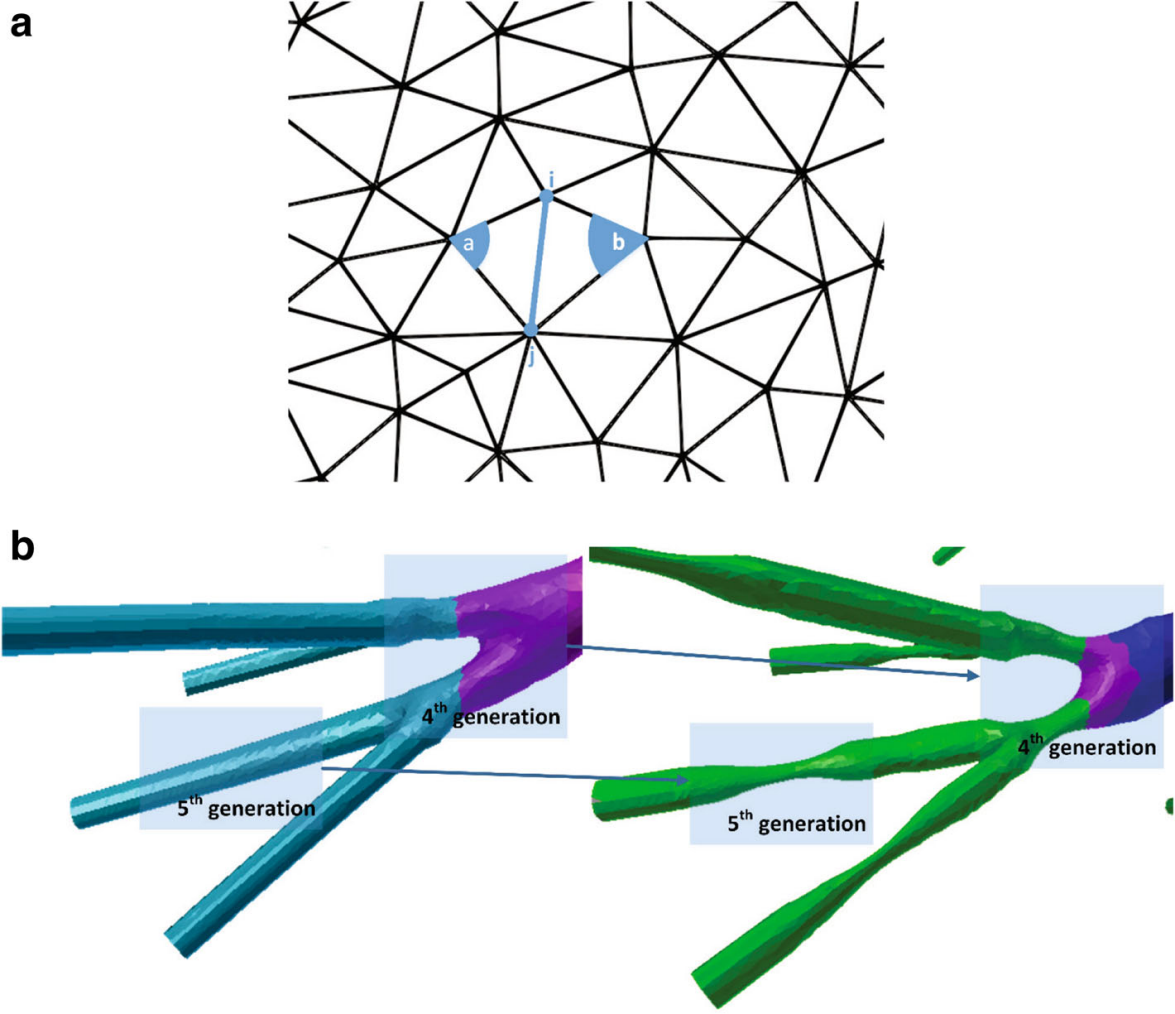
Geometry of obstructed airways related to the 4th and 5th generation: (**a**) perspective of surface mesh and geometrical parameters and (**b**) narrowing is employed by a percentage of 50%

The proposed lung geometry contraction procedure builds upon the method presented in [32] enabling a unique geometry processing methodology. This technique suggests removing details and noise from the mesh surface by employing a Laplacian smoothing process that shifts the vertices along their estimated curvature normal direction, as shown in Fig. 2. Initially, we present a short overview of the method that uses an iterative updating scheme regulated by anchor points that serve as positional constraints. To be more specific, the vertex positions **V** are smoothly diminished along the normal direction by iteratively solving the following minimization problem

2 $$ {\tilde{\boldsymbol{V}}}^{\prime }={argmin}_{\boldsymbol{V}}\left({\left\Vert {\boldsymbol{W}}_L\boldsymbol{LV}\right\Vert}^2+{\boldsymbol{W}}_H\left\Vert \boldsymbol{V}-{\boldsymbol{V}}_a\right\Vert \right) $$

where *V*_*aj*_ = *V*_*j*_,  *j* = 1, . . , *N*, **V** is the vector with the vertices of the mesh, *N* is the number of vertices of the mesh, *V*_*j*_ is the vertex with index *j* before the contraction initiates and **L** is the *n* × *n* curvuture flow Laplacian operator with elements:

3 $$ {\boldsymbol{L}}_{i,j}=\left\{\begin{array}{cc}{\omega}_{i,j}={cota}_{i,j}+{cotb}_{i,j}& \left(i,j\right)\in E\\ {}\sum \limits_{i,k\in E}^k-{\omega}_{ik}& i=j\\ {}0& otherwise\end{array}\right. $$

where *a*_i,j_, *b*_i,j_ denote the angles facing the edge (i,j). These angles are included to the adjacent triangles that surround the edge (i,j), as illustrated in Fig. 2(a). Since **L** is singular, further anchor constraints are used to ensure a unique solution for **V** ^′^, attracting the vertices to the geometry.

The solution of the aforementioned optimization problem is obtained by solving the following system of equations:

4 $$ \left[\begin{array}{c}{\boldsymbol{W}}_L\boldsymbol{L}\\ {}{\boldsymbol{W}}_H\end{array}\right]{\boldsymbol{V}}^{\prime }=\left[\begin{array}{c}\mathbf{0}\\ {}{\boldsymbol{W}}_H\boldsymbol{V}\end{array}\right] $$

where **W**_*L*_ and **W**_*H*_ are diagonal matrices. The analytical solution can be expressed as:

5 $$ {\tilde{\boldsymbol{V}}}^{\prime }={\left({\boldsymbol{A}}^T\boldsymbol{A}\right)}^{-1}\boldsymbol{Ab} $$

where $$ \mathbf{A}=\left[\begin{array}{l}{\mathbf{W}}_L\mathbf{L}\\ {}{\mathbf{W}}_H\end{array}\right] $$ and **b**=$$ \left[\begin{array}{l}\mathbf{0}\\ {}{\mathbf{W}}_H\mathbf{V}\end{array}\right] $$.

In order to collapse the entire model into an 1D shape, various iterations with proper weights are required. After the first iteration, specific high-frequency details are sorted out and the mesh is remarkably contracted. Nonetheless, the utilization of the same weights *W*_*L*_ and *W*_*H*_ in subsequent iterations does not result to any further contraction. Therefore, to increase the collapsing speed, we augment the contraction weight *W*_*L*,*i*_ for every vertex *i* after each iteration.

**Fig. 3:**
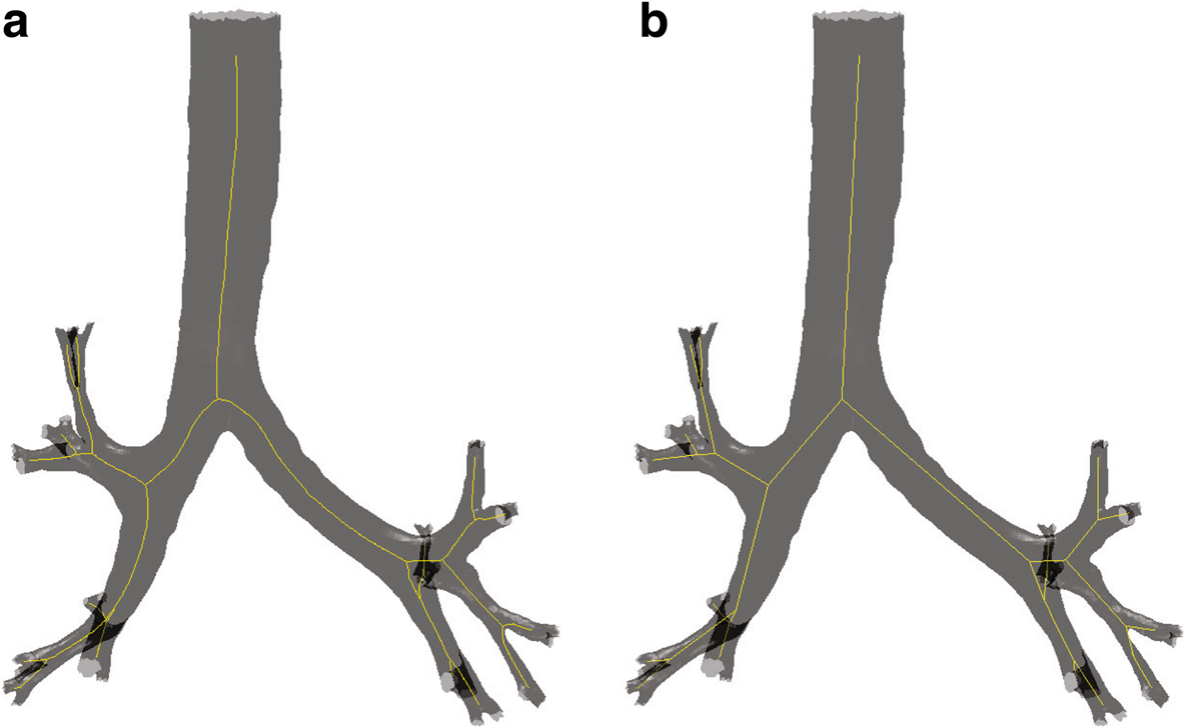
1D representation of the patient personalized model of lung geometry: (**a)**) central pathway and (**b**) linearized pathway

In order to simplify the geometry and extract the 1D representation as described in [31] a surgery connectivity procedure is required. The resulting mesh consists of connected nodes that are connected to either to one, two or three nodes. For further simplification, the adjacent nodes are merged into new ones and the connections between them are eliminated. Figure 3 depicts a simplified 1D representation of the 3D geometry.

Moreover, a fine tuning of the contraction weights is required to enhance the mesh contraction procedure and to achieve a specific narrowing ratio. For this purpose, we intoduce a new weighting diagonal matrix ${\mathbf{W}}_L^{0^{\prime }}$ employing the following formulation:

6 $$ {\boldsymbol{W}}_L^{0^{\prime }}=\omega \cdotp {\boldsymbol{W}}_L^0 $$

where *ω* is a scalar value and 0 ≤ *ω* ≤ 1.

#### Termination criteria

The geometry contraction process is an iterative process that requires termination criteria to be established. In the case that the geometry collapses to an 1D shape, the process is repeated until the mesh volume is less than 10^−7^. In any other case, we need to estimate the degree of contraction of the airway’s geometry after each iteration. For this purpose, we employ a shape diameter function (SDF) based scheme [32] that evaluates the local volume based on the estimated local diameter assigned to each face of the mesh, also known as raw SDF values.

##### Computing raw SDF values

For a specific point on the surface mesh a cone is utilized being centered around its inward-normal direction (the opposite direction of its normal). Numerous rays are sent inside this cone to the other side of the mesh. For each ray of this kind, a check is employed for the normal at the point of intersection, while we ignore any intersection where the normal at the intersection points appears in the same direction as the point-of-origin (the identical direction is described as an angle difference less than 90^∘^). In this way, fake intersections with the outer part of the mesh are removed. The SDF at a point is denoted as the weighted average of all lengths of the rays, which fall within one standard deviation from the median of all lengths. The weights employed are the inverse of the angle between the ray to the center of the cone. This is explained by the fact that rays with greater angles are more frequent, and thus have reduced weights.

##### Post-processing of raw SDF values

After having calculated the raw SDF value for each facet, the SDF values that can be used in the segmentation algorithm are the result of several post-processing steps:

- Facets with no raw SDF values are assigned the average raw SDF value of their edge-adjacent neighbors.
- A bilateral smoothing [33] is applied. This smoothing technique removes the noise while trying to keep fast changes on SDF values unchanged, since they are natural candidates for segment boundaries. The bilateral smoothing has three parameters that are set as follows:
  - $w=\left\lfloor \sqrt{\mid F\mid /2000}\right\rfloor +1$, the window size (i.e. maximum level for breadth-first neighbor selection), where *F* denotes the set of facets
  - *σ*_*s*_ = *w*/2, the spatial parameter
  - ${\sigma}_{r_i}=\sqrt{1/\mid {w}_i\mid \sum \limits_{f_j\in {w}_i}{\left( SDF\left({f}_j\right)- SDF\left({f}_i\right)\right)}^2}$, the range parameter set for each facet *f*_*i*_ and *w*_*i*_denotes the set of neighboring facets of *f*_*i*_collected using *w* in the facet neighbor breadth-first search
- SDF values are linearly normalized between [0, 1].

##### Soft and hard clustering

Given a number *k* of clusters, the soft clustering is a Gaussian mixture model that consists in fitting *k* Gaussian distributions to the distribution of the SDF values of the facets. It is initialized with k-means++ [34], and run multiple times with random seeds. Among these runs, the best result is used for initializing the expectation-maximization algorithm for fitting the Gaussian distributions.

The hard clustering yields the final segmentation of the input mesh and results from minimizing an energy function combining the aforementioned probability matrix and geometric surface features. The energy function minimized using alpha-expansion graph cut algorithm [35] is defined as follows:

7 $$ E\left(\overline{x}\right)=\sum \limits_{f\in F}{e}_1\left(f,{x}_f\right),+\lambda \sum \limits_{\left\{f,g\right\}\in N}{e}_2\left({x}_f,{x}_g\right) $$

8 $$ {e}_1\left(f,{x}_f\right)=-\mathit{\log}\left(\mathit{\max}\left(P\left(\left.f\right|{x}_f\right),\varepsilon \right)\right) $$

9 $$ {e}_2\left({x}_f,{x}_g\right)=\left\{\begin{array}{cc}-\mathit{\log}\left(\theta \left(f,g\right)/\pi \right)& {x}_f\ne {x}_g\\ {}0& {x}_f={x}_g\end{array}\right\} $$

where *F* denotes the set of facets, *N* denotes the set of pairs of neighboring facets, *x*_*f*_ denotes the cluster assigned to facet *f*, *P*(*f*| *x*_*p*_) denotes the probability of assigning facet *f* to cluster *x*_*p*_, *θ*(*f*, *g*) denotes the dihedral angle between neighboring facets *f* and *g* : concave angles and convex angles are weighted by 1 and 0.1 respectively, *ε* denotes the minimal probability threshold, *λ* ∈ [0, 1] denotes a smoothness parameter.

##### Deriving airway narrowing ratio

The SDF values assigned to each face correspond to the local diameter of the shape. Hence, by summing up the SDF values of the faces of the processed segment of the mesh we extract a representative value for the local mean diameter of the processed part. The ratio *r* ^*t*^ of the sum of the SDF values before the narrowing procedure to the sum of the SDF values after the narrowing procedure is associated to a metric of the narrowing percentage of the airway

10 $$ {r}^t=\frac{\sum_{i=0}^N SDF\left({f}_i^t\right)}{\sum_{i=0}^N SDF\left({f}_i^0\right)} $$

where *r* ^*t*^ is the narrowing ratio after iteration *t*, ${f}_i^t$ is the face with index *i* after iteration *t*, ${f}_i^0$ is the initial face with index *i* and *N* is the number of processed faces.

##### Bronchoconstriction simulation

Taking into account the aforesaid geometry processing schemes we employ the following algorithm to simulate bronchoconstriction given the desired number of iterations *t*, the user-defined weight *ω*, the desired narrowing ratio *r* and the accepted narrowing ratio error *e*.

1. Initialize **W**_*L*_ and **W**_*H*_ in the following manner:

11 $$ {\boldsymbol{W}}_L=k\cdotp \omega \cdotp \sqrt{A} $$

12 $$ {\boldsymbol{W}}_H=I $$

where **I** is a unitary matrix, k a double constant set to 10^−3^ and A the average face area of the model.

2. Solve (2) for **V** ^′^.
3. Update **W**_*L*_ and **W**_*H*_ so that

13 $$ {\boldsymbol{W}}_{\boldsymbol{L}}^{\boldsymbol{t}+1}={s}_L\cdotp {\boldsymbol{W}}_{\boldsymbol{L}}^{\boldsymbol{t}} $$

14 $$ {\boldsymbol{W}}_{\boldsymbol{H},\boldsymbol{i}}^{\boldsymbol{t}+1}={W}_{H,i}^0\cdotp \sqrt{A_i^0/{A}_i^t} $$

where t represents the iteration index.

4. Recompute **L**.
5. Compute narrowing ratio *r* ^*t*^.
6. Repeat steps 2 to 5 *r* ^*t*^ > *r* + *e*.

**Fig. 4:**
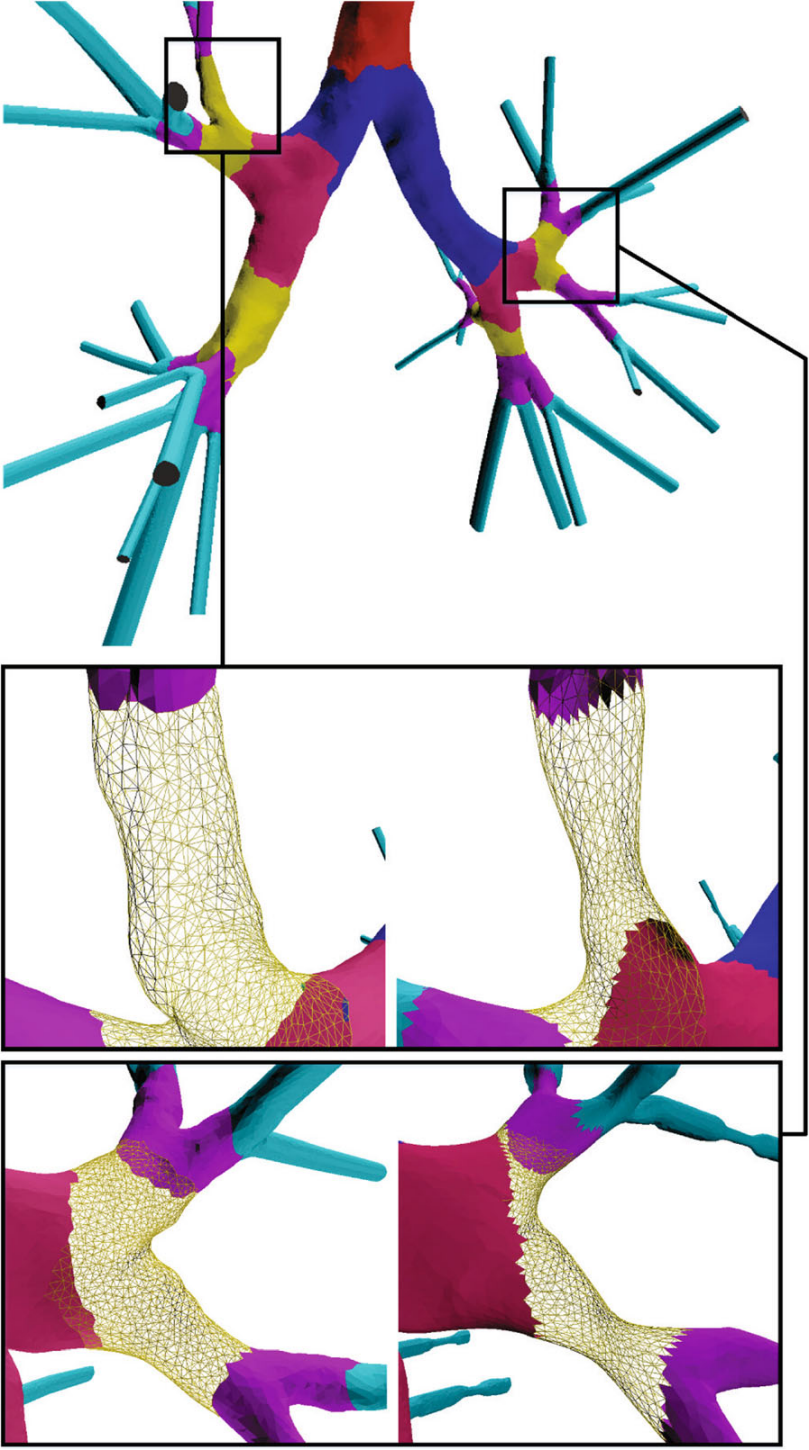
Geometry of obstructed airways related to the 3rd generation. Magnified perspective before and after the narrowing process

For the current investigation, we set *r* = 0.5, *e* = 0.05, *t* = 8 and *ω* = 0.8. The area under study is segmented for more efficient processing and mesh contraction is performed. Figure 2(b) illustrates the bronchoconstriction simulation for airways of the 4^*th*^, and 5^*th*^ generation, whereas Fig. 4 depicts a magnified perspective of the 3^*rd*^ generation before and after the narrowing process. These cases exhibit a narrowing ratio of 50%.

##### Surface abnormalities simulation

The application of different levels of deformation across the airways as a function of distance is proposed in order to enhance the mesh contraction procedure and to simulate the surface abnormalities during bronchoconstriction. Therefore, as the authors in [36] propose, we set a new ${\mathbf{W}}_L^{0^{\prime }}$ weighting diagonal matrix employing the following formulation:

15 $$ {\boldsymbol{W}}_L^{0^{\prime }}={\boldsymbol{W}}_L^0\cdotp {\boldsymbol{W}}_V $$

where **W**_*V*_ is a diagonal weighting matrix. For **W**_*V*_ we can use a custom function:

16 $$ {\boldsymbol{W}}_V=f(d) $$

where *d* is the geodesic distance of a point of the mesh from an anchor point.

A sinusoidal function is utilized in this study for demonstration purposes only. The user can define the function on a case-by-case basis. Therefore, a weighting parameter is set for each vertex equal to:

17 $$ {\boldsymbol{W}}_V=\omega +\frac{1-\omega }{2}\cdotp sind $$

where *ω* is the user defined parameter.

##### Mitigation of edge effects

Discontinuities and edge effects may appear considering that the processed part is reconnected with the rest of the mesh. To deal with this issue, we employ surface smoothing methods such as Taubin smoothing [37] or bilateral surface denoising at the part of the mesh under process taking into account the edge points (anchor points) of the stable area of the mesh.

**Fig. 5:**
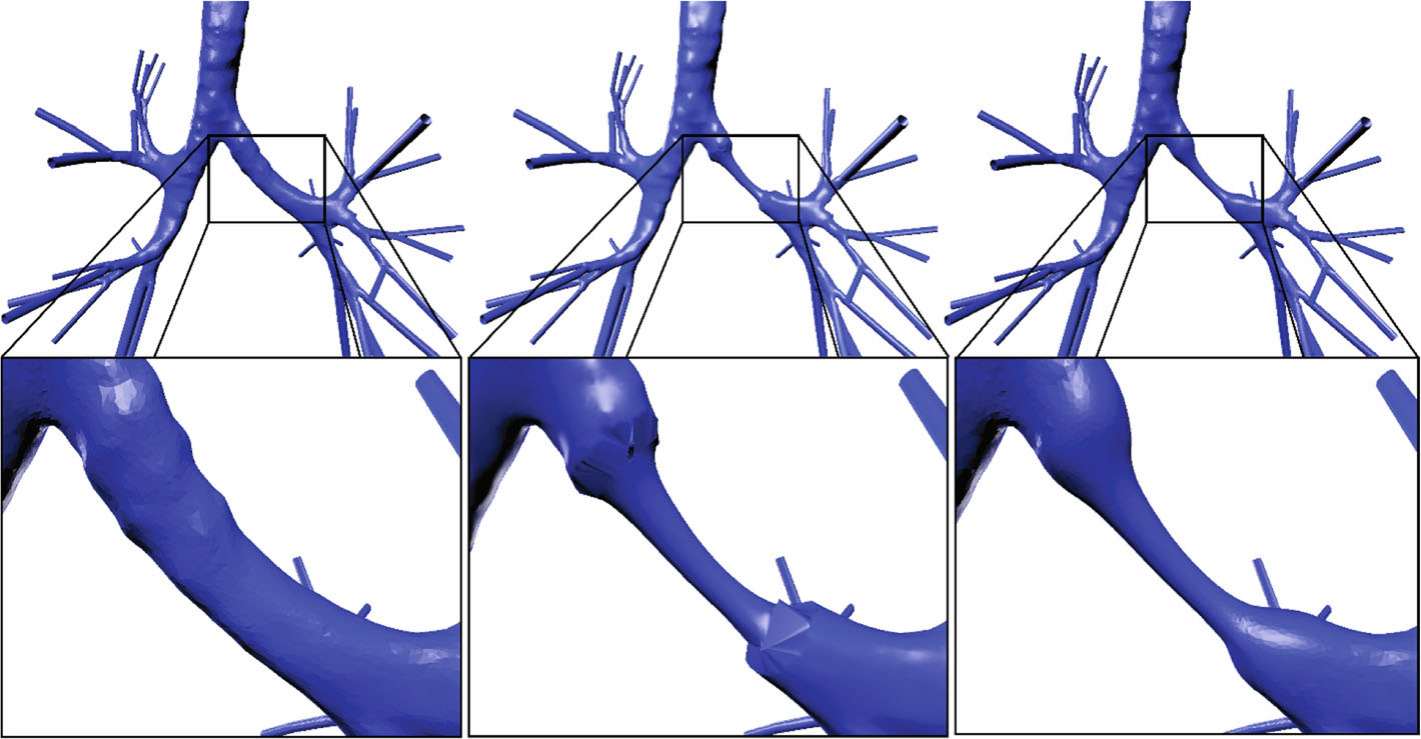
Procedure of addressing edge effects. Magnified perspective at various steps of the narrowing process

The bilateral technique estimates the new normals that can be used for vertex updating according to:

18 $$ {\overline{\boldsymbol{n}}}_m={\left({\boldsymbol{D}}^{-1}{\boldsymbol{C}}_w\right)}^{\zeta }{\widehat{\boldsymbol{n}}}_n $$

where **D** is a diagonal matrix, *ζ* is an integer that represents a denoising factor and

19 $$ {\boldsymbol{C}}_w={\boldsymbol{W}}_m{\boldsymbol{W}}_n{\boldsymbol{C}}_a $$

is the weighted bilateral adjacency matrix. Eq. (18) shows that any averaging filter on the normals, is a frequency selective transform. For every centroid **m**_*i*_ ∀ *i* = 1, ⋯, *n*_*f*_ the weights of ${\mathbf{W}}_m,{\mathbf{W}}_n\in {\mathfrak{R}}^{n_f\times {n}_f}$ are estimated according to the following equations:

20 $$ {\boldsymbol{W}}_{m_{ij}}=\left\{\begin{array}{ll}\mathit{\exp}\left(-{\left\Vert {\boldsymbol{m}}_i-{\boldsymbol{m}}_j\right\Vert}^2/2{\sigma}_m^2\right)& if\;{\boldsymbol{C}}_{a_{ij}}=1\\ {}0& otherwise\end{array}\right. $$

21 $$ {\boldsymbol{W}}_{n_{ij}}=\left\{\begin{array}{ll}\mathit{\exp}\left(-{\left\Vert {\widehat{\boldsymbol{n}}}_{mi}-{\widehat{\boldsymbol{n}}}_{mj}\right\Vert}^2/2{\sigma}_n^2\right)& if\;{\boldsymbol{C}}_{a_{ij}}=1\\ {}0& otherwise\end{array}\right. $$

where **C**_*a*_ is the binary adjacency matrix constructed using the k-nearest neighbors method. Finally, the vertices in each iteration *t* are updated according to:

22 $$ {\boldsymbol{v}}_i^{\left(t+1\right)}={\boldsymbol{v}}_i^{(t)}+\frac{\sum_{j\in {\boldsymbol{\varPsi}}_i}{\overline{\boldsymbol{n}}}_{mj}^{(t)}\left(<{\overline{\boldsymbol{n}}}_{mj}^{(t)},\left({\boldsymbol{m}}_j^{(t)}-{\boldsymbol{v}}_i^{(t)}\right)>\right)}{\mid {\boldsymbol{\varPsi}}_i\mid } $$

23 $$ {\boldsymbol{m}}_j^{\left(t+1\right)}=\left({\boldsymbol{v}}_{j1}^{\left(t+1\right)}+{\boldsymbol{v}}_{j2}^{\left(t+1\right)}+{\boldsymbol{v}}_{j3}^{\left(t+1\right)}\right)/3\kern1em \forall \kern0.5em j\in {\boldsymbol{\varPsi}}_i $$

where <**a**, **b**> represents the inner product of **a** and **b**, (*t*) represents iteration number and **Ψ**_*i*_ represents the set of the face vertices which are connected with **v**_*i*_. Equations (18), (22) and (23) are executed iteratively for a given number of iterations until a certain reconstruction quality criterion is met. Figure 5 shows the edge effects before and after the smoothing process.

### Physical modeling of the lung

Various methods, such as surface reconstruction, surface remeshing, triangulation, and decimation are employed for cleaning-up the lung geometry, in order to enable smoothness and accelerate the upcoming techniques of volume meshing and solving. A smoothing process is considered compulsory to minimize any adverse effects on the study results, since the CFD analysis could be affected by additional edge effects created by the deformation of the airway geometry. Furthermore, our analysis will not be disturbed much by the structural modeling procedures, since the airflow into the lungs is essentially resolved by the topology of the generations’ geometry rather than the surface arrangement of the airways.

An open source platform associated to CFD and FPT analyses, called OpenFoam [38], is efficiently employed, in order to implement the volume meshing of the lung by utilizing the snappyHexMesh algorithm. A very fine discretization near the walls of the pulmonary system is adopted by our analysis that involves hex-dominant cells. In this manner, the stimulating properties of turbulence can be discerned, while the positions of particles can be precisely determined. Specifically, the domain is discretized into 5,569,106 cells, which denote a huge computational burden. In order to effectively tackle this issue, ARIS, a high performance computer (HPC), was utilized to conduct the involved simulations.

#### CFD analysis of airway system

The lung’s activity can be computationally evaluated by employing various models, such as Reynolds-averaged Navier-Stokes (RANS) or Reynolds-averaged simulation (RAS), direct numerical simulation (DNS) and large eddy simulation (LES). Amid them, the RAS model exhibits acceptable levels of accuracy along with the minimum computational cost [18]. In this context, RAS is effectively utilized to conduct a steady state CFD analysis of the human respiratory system by solving the Navier-Stokes equations. Specifically, the properties of the airflow are clarified by employing a kOmegaSST turbulence model. The investigation is performed through the FVM algorithm, particularly the SIMPLE technique, which is part of the OpenFoam platform. Second order schemes in time and space are applied to perform the discretization of the associated eqs. A time step of 5 × 10^−4^ s is employed in order to allow numerical stability. The Courant number, the air velocity, and the mesh discretization, as designated in the CFD theoretical formulations, determine the value of the aforementioned time step. In this manner, stability and convergence of the simulations are ensured. A hypothesis of a total pressure drop of −15 Pa is taken into account throughout the simulations of inspiratory states. An additional evaluation of the inhaled particles attributes is allowed by the airflow velocity and pressure, which are assessed over the computational domain.

#### FPT analysis of inhaled particles

The motion of particles in fluids is examined by utilizing a Lagrangian approach that solves a set of ordinary differential equations along their trails. The modification of the particle location as well as the components of the particle velocity are extracted by the fluid particle tracing procedure. The associated forces applied on the particle need to be considered. Consequently, the differential equations for determining the particle location and velocity are specified by Newtonian second law, if spherical particles are taken into account:

24 $$ \frac{d{x}_P}{dt}={u}_P $$

25 $$ {m}_P\frac{du_P}{dt}=\sum {F}_i $$

26 $$ {I}_P\frac{d{\omega}_P}{dt}=T $$

where ${m}_P={\rho}_P{d}_P^3\pi /6$ is the mass of a particle (*ρ*_*P*_ is the density of a particle and *d*_*P*_ is the diameter of a particle), ${I}_P=0.1{m}_P{d}_P^2$ is the moment of inertia for a sphere, *F*_*i*_ represents the relevant forces applied on the particle, *u*_*P*_ is the linear velocity of a particle, *ω*_*P*_ is the angular velocity of a particle and *T* is the torque applied on a rotating particle due to the viscous interaction with the fluid.

Analytical solutions are offered for the distinct forces taken into account (Stokes flow) for small Reynolds numbers. A coefficient *C* is introduced to be multiplied with the force, in order to adopt an extension to higher Reynolds numbers. This coefficient *C* is based on empirical correlations obtained from direct numerical simulations or experiments. Moreover, the drag force dictates the particle’s motion in most fluid-particle systems. A drag coefficient *C*_*D*_ is introduced as an extension to higher particle Reynolds number, whereas it is defined from

27 $$ {C}_D=\frac{F_D}{\frac{\rho_F}{2}{\left({u}_F-{u}_P\right)}^2{A}_P} $$

where *u*_*F*_ is the linear velocity of a fluid, and ${A}_P={d}_P^2\pi /4$ is the cross-section of a spherical particle. The drag force can be described by:

28 $$ {F}_D=\frac{3}{4}\frac{\rho_F{m}_P}{\rho_P{d}_P}{C}_D\left({u}_F-{u}_P\right)\left|{u}_F-{u}_P\right| $$

The ratio of inertial force to friction force specifies the particle Reynolds number as:

29 $$ {\mathit{\operatorname{Re}}}_P=\frac{\rho_F{d}_P\left|{u}_F-{u}_P\right|}{\mu_F} $$

where *ρ*_*F*_ is the density of the fluid and *μ*_*F*_ is the dynamic viscosity of the fluid. The aforesaid methodology is intended to allow the evaluation of the particle deposition into the respiratory system. In addition, it is envisaged as the first step for the detailed analysis of drug delivery efficiency, whereas it enables conclusions related to the selection of medication (size and concentration of particles).

The transfer of inhaled particles through the pulmonary system, along with the deposition fraction over several areas of interest, are investigated by a transient FPT analysis. In particular, the steady state field of airflow velocity obtained by the CFD simulation is employed to estimate the position and velocity of the particles, because of the one-way coupling between the flow field and the particles that is taken into consideration. Our simulations involve the density of the particles that is selected to be 1000.0 kg/m^3^, whereas their diameter is set to 10 μm, taking into account that typical coarse ambient particles (PM10) are 2.5 μm to 10 μm in diameter. Moreover, the flowrate of air at the normal lung model is 12.6 L/min, which allows particles with a diameter of 10 μm to be transported into the central generations. The inlet boundary of the computational domain is considered the input of the system, since a total of 54,000 particles are injected through it.

The initial velocity of the particles as well as the inlet velocity of air for the CFD simulations are deemed the same. For the normal lung model, the inlet velocity of air is 1.475 m/s, which corresponds to a flowrate of 12.6 L/min. In addition, the substance of the particles is injected during an injection period of 0.75 msec by a flowrate of 199.2 mL/min to allow accurate identification of the deposition patterns. An effective simulation of the particle’s entrapment on the wall’s surface is accomplished by a stick boundary condition which is employed on the wall of the lung topology as well as on the outlets. The icoUncoupledKinematicParcelFoam solver, which is part of the OpenFoam platform, is utilized to conduct a transient analysis. In this method the flow affects the particles but the particles don’t affect the flow, since it presumes that the particles’ effects on turbulence are negligible. Consequently, an estimation of the position of the particles on the pulmonary airways is attained.

## Results

### Numerical evaluation of the respiratory system

Various lung models related to diverse levels of narrowings have been generated using the method previously designated. More specifically and in addition to the unobstructed lung model, a group of obstructed models have been created. Each model is characterized by the lung generation after which narrowings with a structure similar to that of a bottleneck are introduced. Furthermore, an asymmetric case where obstructions are introduced only in the right lung after the second generation is examined. An in-house established software, based on the aforesaid geometry processing sequences, have been effectively utilized for the development of these models. A CFD analysis is conducted for each model, while the output velocity fields are exploited for the corresponding FPT investigations. As a next step, the calculation of the particles deposition is attained for every segment of the lung’s model. In this context, deposition fractions are obtained for each generation of the pulmonary system, besides the left/right sections.

#### CFD analysis

**Fig. 6:**
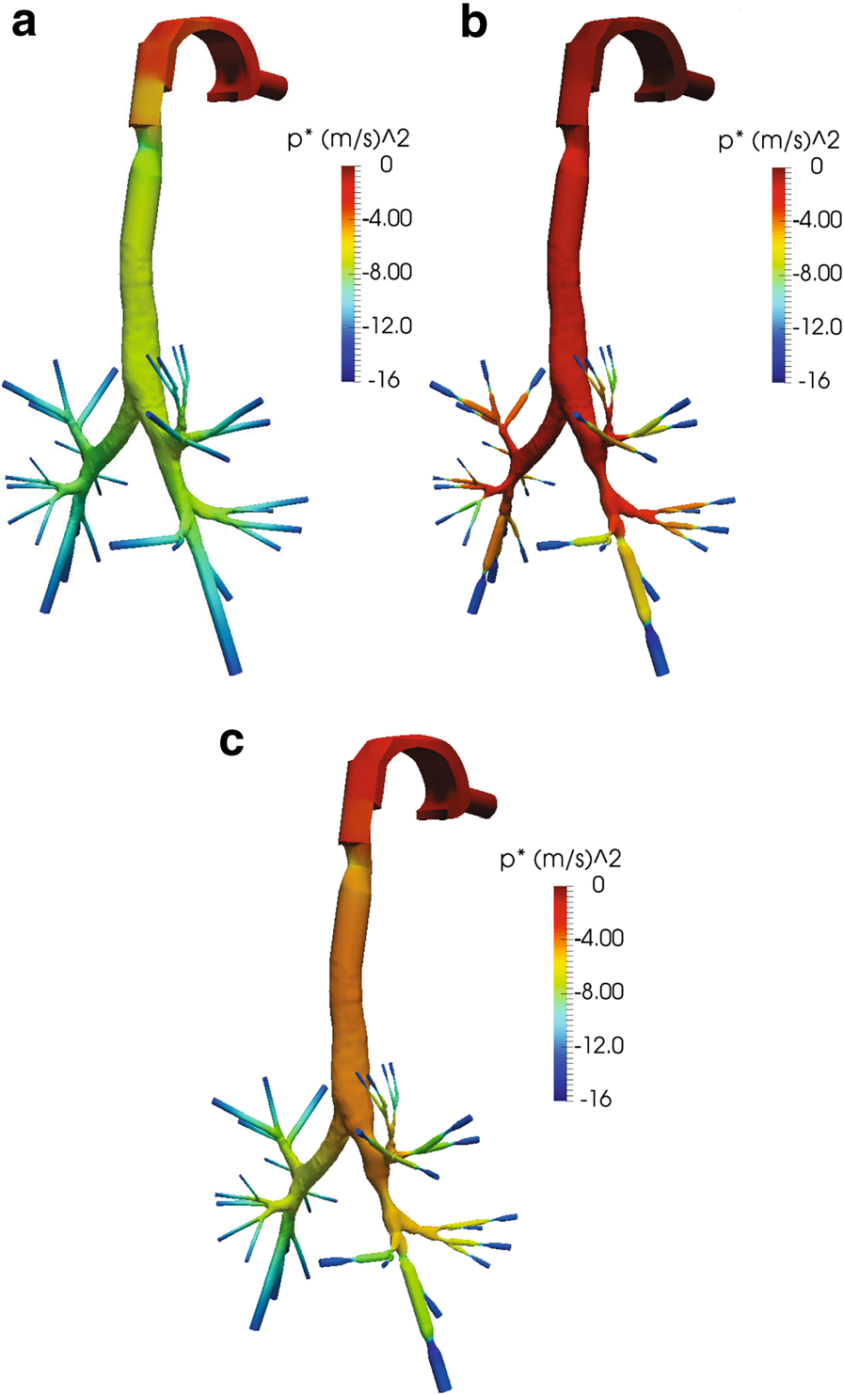
Normalized pressure drop (m^2^/s^2^) at the surface of the lung for various cases: (**a**) normal pulmonary system (**b**) obstructions in both lungs and (**c**) obstructions in right lung only

**Fig. 7:**
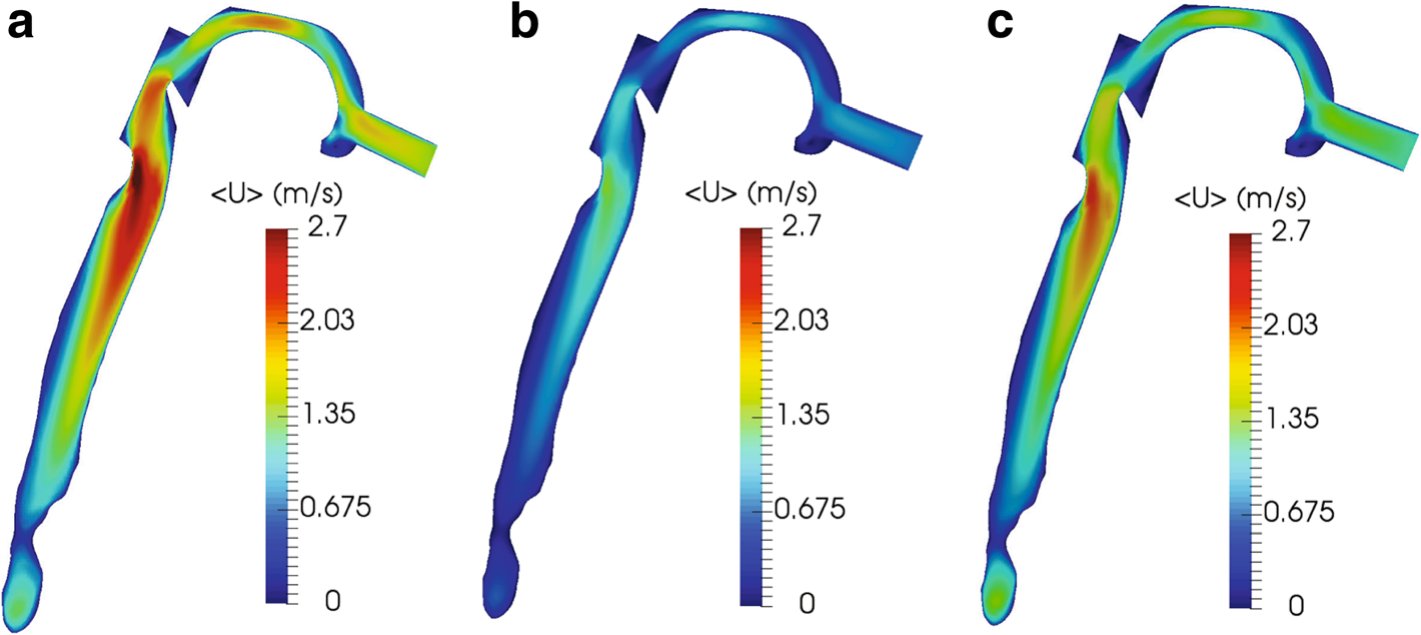
Air velocity (m/s) at the sagittal plane cross-section of the upper respiratory tract for various cases: (**a**) normal pulmonary system (**b**) obstructions in both lungs and (**c**) obstructions in right lung only

**Fig. 8:**
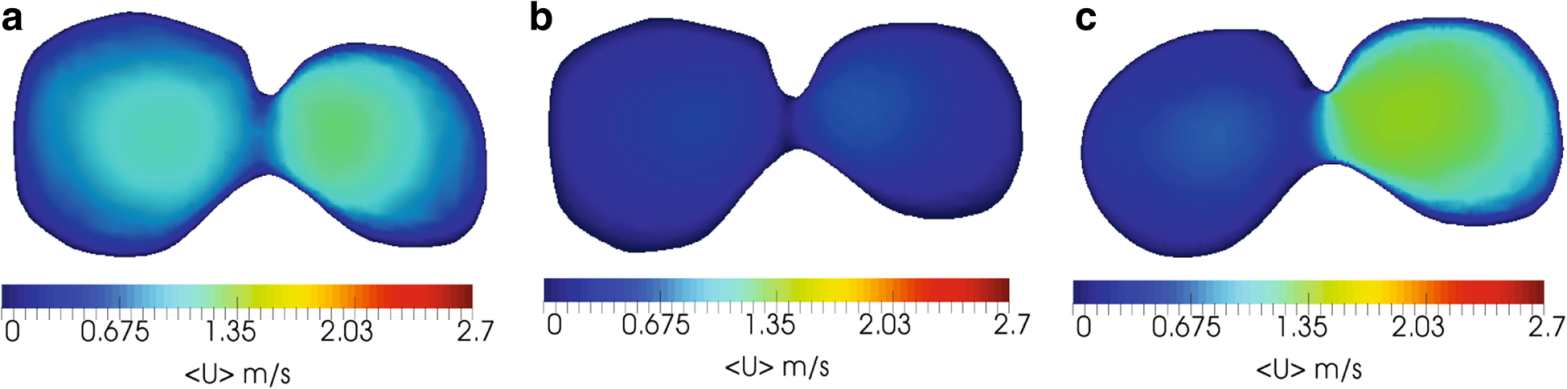
Air velocity (m/s) at the transverse cross-section at the airways main bifurcation for various cases: (**a**) normal pulmonary system (**b**) obstructions in both lungs and (**c**) obstructions in right lung only

**Fig. 9:**
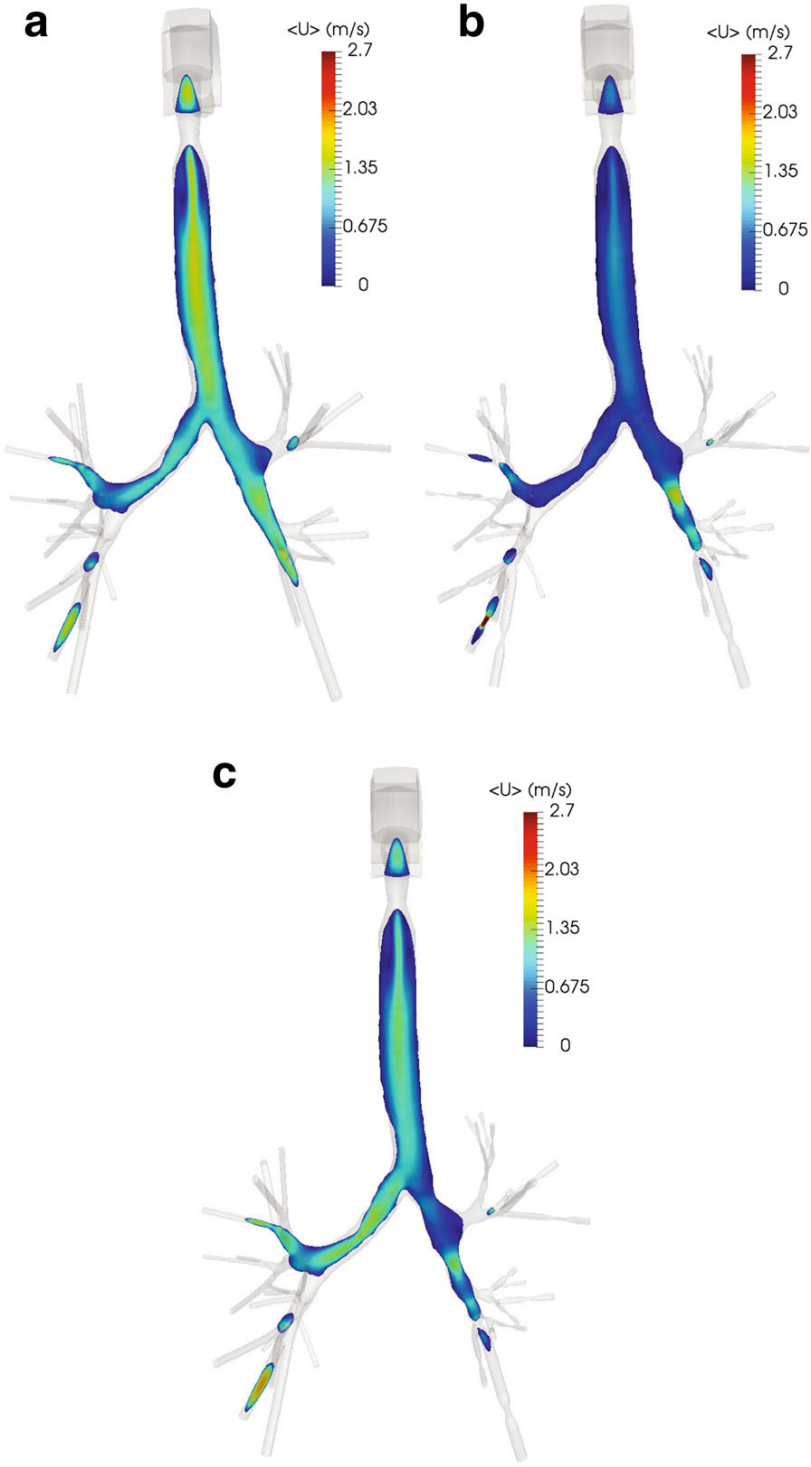
Air velocity (m/s) at the coronal plane cross-section at the trachea and main bronchi for various cases: (**a**) normal pulmonary system (**b**) obstructions in both lungs and (**c**) obstructions in right lung only

A total pressure drop of −15 Pa is presumed in all cases as certified by past studies of airflow into the human respiratory system [16, 22], thus the inlet velocity of the air is defined by the simulations. For instance, the normal lung demonstrates an inlet velocity of 1.475 m/s and a flowrate of 12.6 L/min, whereas the velocity of air when narrowings are detected after the second generation is 0.711 m/s and the flowrate is 6.06 L/min. Also, when obstructions occur in the right lung only, an inlet velocity of 1.311 m/s and a flowrate of 11.2 L/min are observed. Indicative results are illustrated in Figs. 6, 7, 8 and 9, where the normalized pressure drop at the surface of the lung, the air velocity distribution cross-sections at the sagittal plane, the transverse plane, the trachea, and main bronchi, are presented. From the results depicted in Fig. 6, higher values of pressure drop are observed on the trachea and the main bronchi, when obstructions in both lungs occur. As observed from Fig. 7, when several narrowings are present, the airflow is decreased at the region of the trachea, thus diminishing the breathing capability. On the other hand, the airflow is fairly decreased if only one lung is obstructed. Similar conclusions can be derived from Fig. 8. Furthermore, the airflow features at the region of the main bronchi are clarified in Fig. 9. When only one lung is obstructed, the airflow is driven at the other functional part. This observation designates that any inhaled particles, ambient or medical oriented, will be directed mostly away from the side of the pulmonary system, where the inflammation is more severe.

#### FPT analysis

**Fig. 10:**
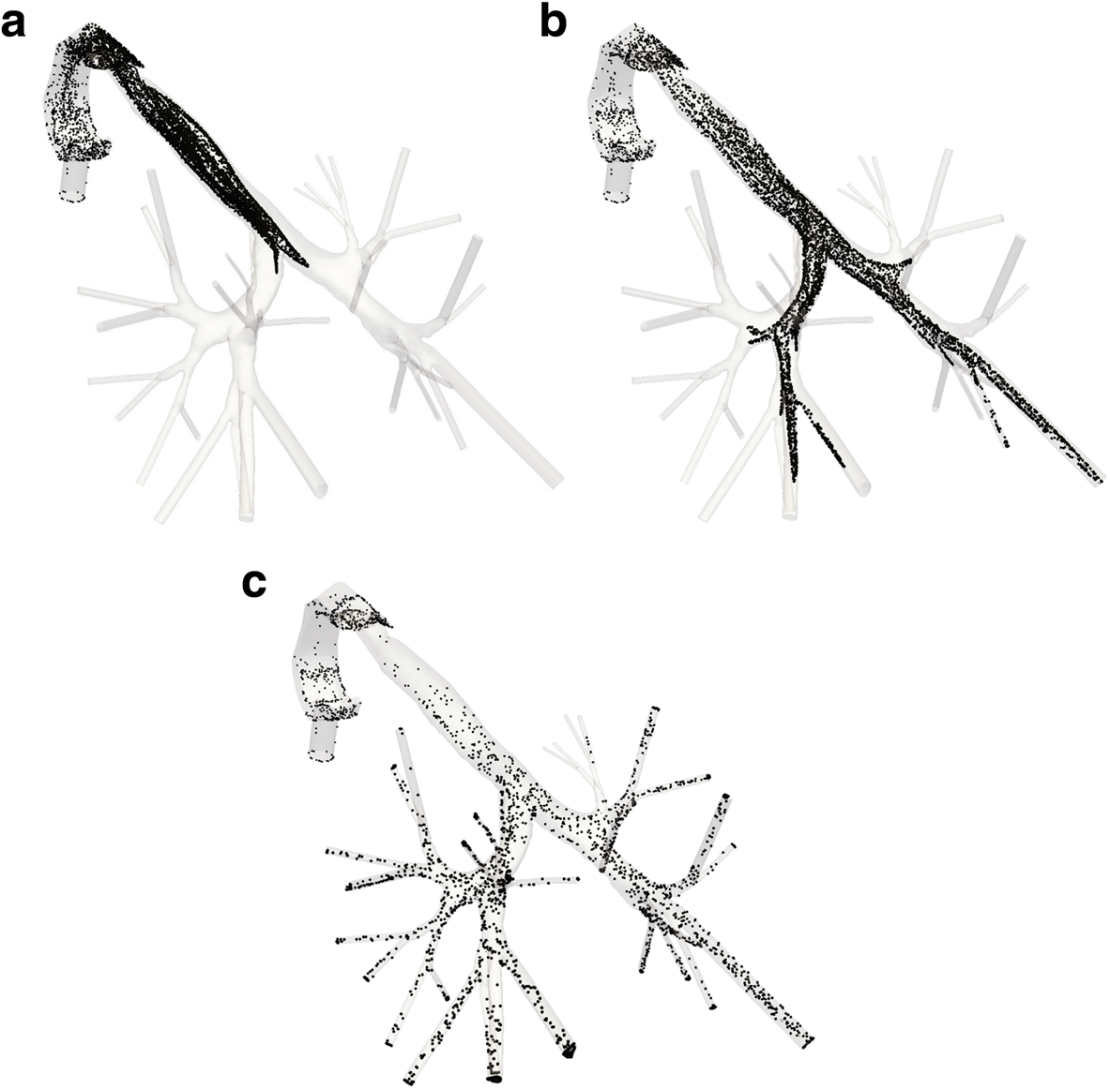
Particles position at the region of the normal lung for distinct time steps during inhalation: (**a**) *t* = 0.2 s (**b**) *t* = 0.3 s and (**c**) *t* = 0.5 s

**Fig. 11:**
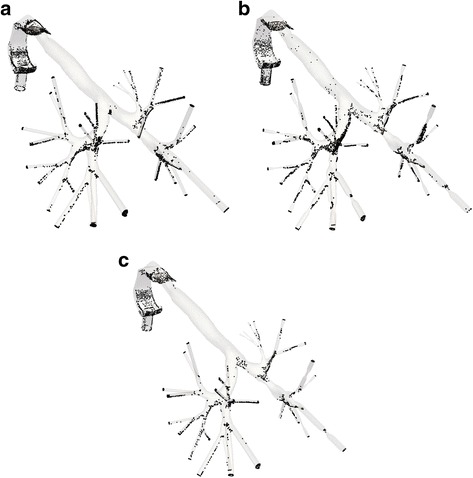
Particles position after the end of the inhalation period for diverse cases: (**a**) normal pulmonary system (**b**) obstructions in both lungs and (**c**) obstructions in right lung only

For each lung model examined in the previous paragraph, a transient analysis of the particles characteristics is performed. A total duration of 2.16 s is considered in order to simulate the whole inhalation procedure. Three snapshots of the particles transfer into the domain of the normal lung are depicted in Fig. 10 for indicative time steps. The particles will stick on distinct regions of the lung, depending on the diverse conditions and obstruction arrangements, thus affecting any associated diseases. The deposition of particles after the completion of the inhalation period denotes a more comprehensive perspective of this incident. Fig. 11 illustrates these particles accumulations for three different cases: a normal lung model, a model with obstructions in both lungs and a model with obstructions in the right lung only. As perceived, when narrowings are introduced in both lungs most particles are deposited in the vicinity of obstructions, in comparison to the function of the normal lung. On the contrary and for the case of the model with obstructions in the right lung, the asymmetry leads to reduced DF in the obstructed airways and a respective increased DF in the lung area, where no inflammation was introduced.

**Fig. 12:**
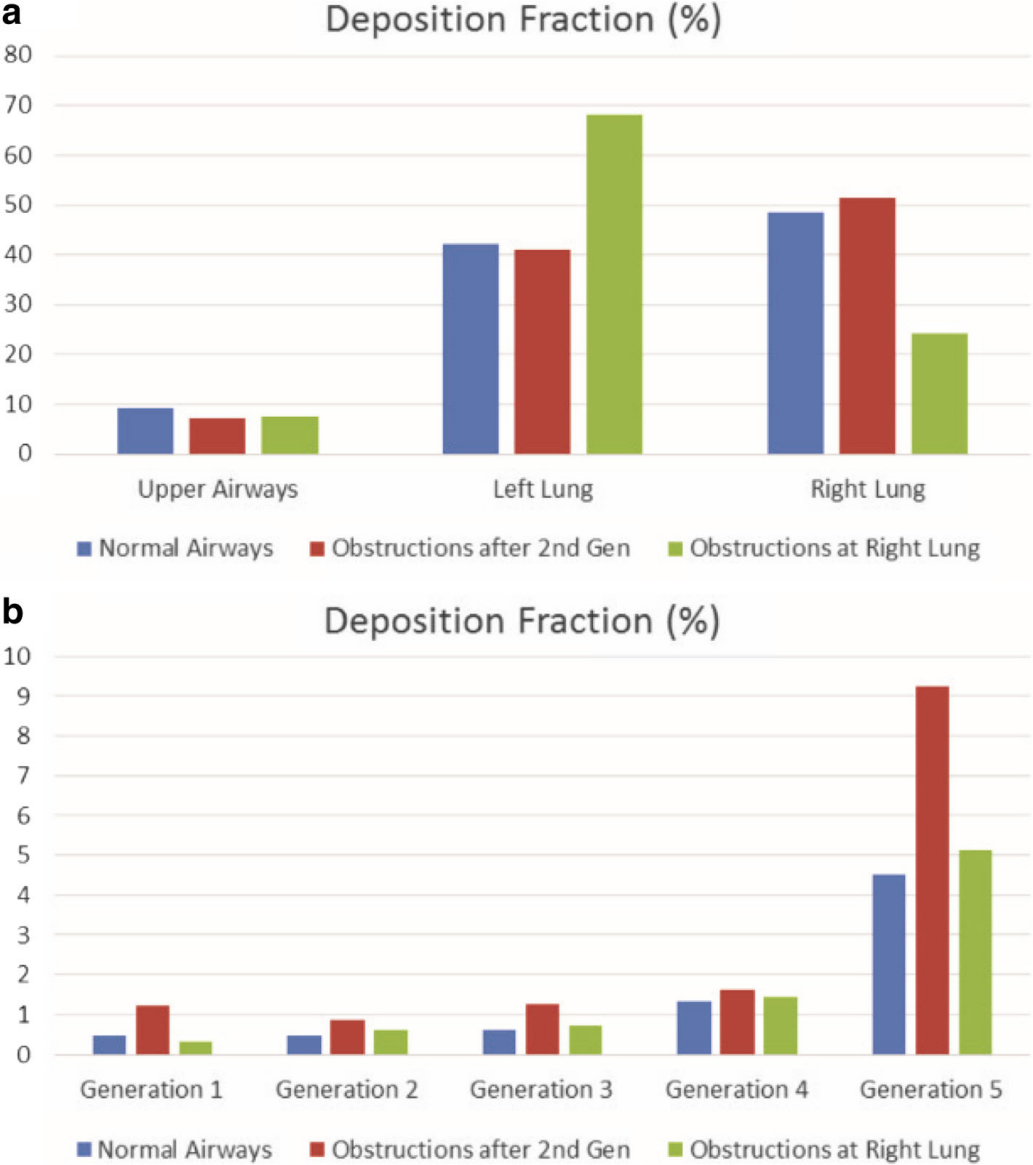
Particles deposition fraction for normal airways, obstructed airways and partially narrowed airways (right lung is obstructed only after the 2nd generation) at: (**a**) various sections of the lungs (including the particles that pass through the outlets) and (**b**) the first five generations. Particle diameter is 10 μm and density is 1000 Kg/m^3^

Moreover, particle DF is acquired for various generations as well as for distinct lung sections. By examining Fig. 12(a), it is deduced that when narrowings occur symmetrically in both lungs the deposition fractions between lungs are slightly affected (41.12% of total for the left lung and 51.45% for the right one). On the other hand, for the air flowrate of 11.2 L/min, most particles are guided on the left lung (68.22% of total), when obstructions are detected on the right section only, as anticipated from the CFD simulations presented in the previous section. In this way, it is indicated that any medication will be delivered away from the problematic blocked region in the case of asymmetric inflammation. This fact should be considered when designing personalized medical treatment of lung maladies. In addition, Fig. 12(b) indicates that the distribution of particles in the 5th generation of airways seems to be almost doubled when obstructions are introduced to both lungs (9.25% of total in comparison to 4.51% of the normal lung). On the contrary, this phenomenon is not so prominent in the case of the model with obstructions only on the right lung.

**Fig. 13:**
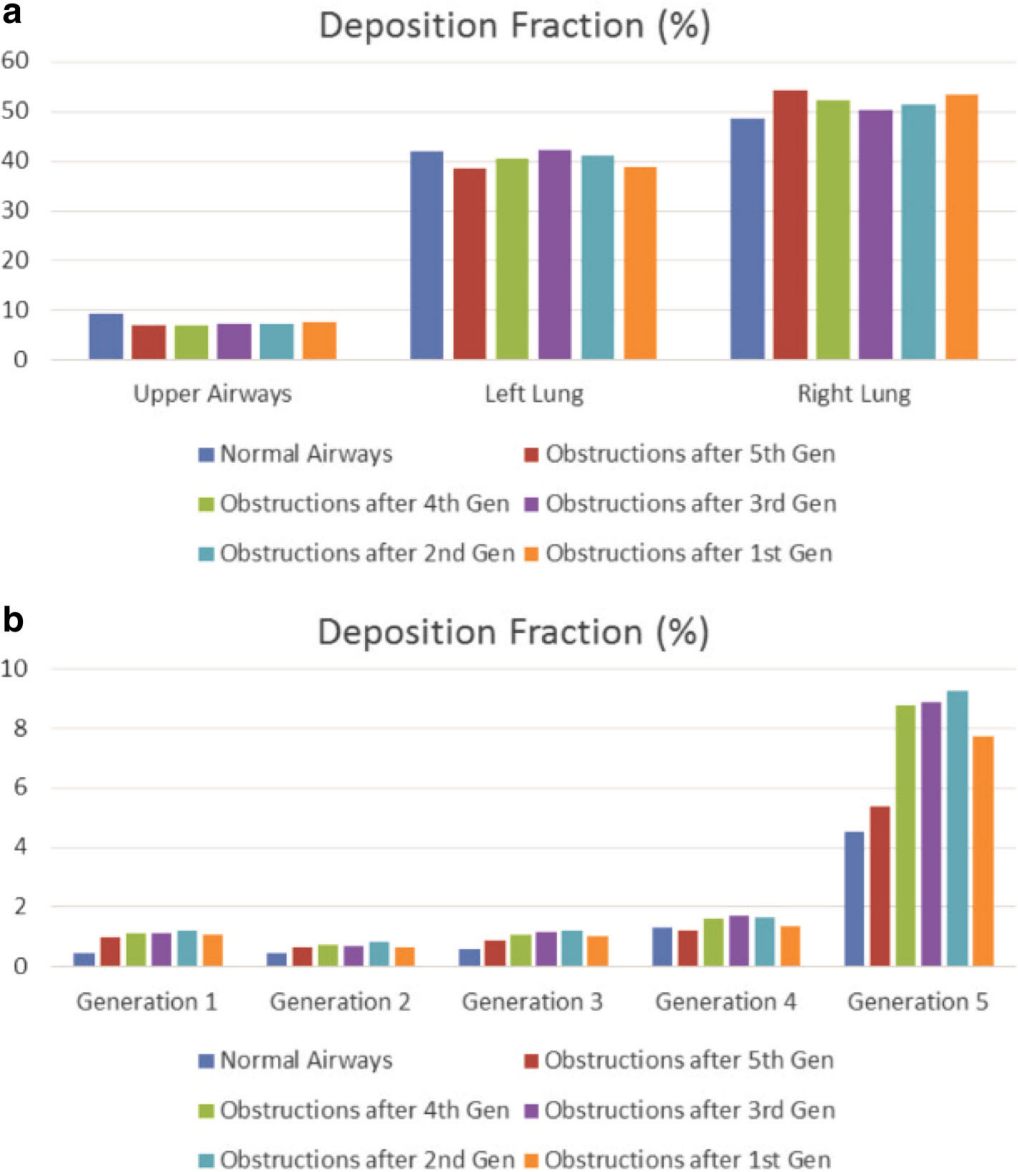
Particles deposition fraction for different lung’s models involving the normal case and diverse obstructions levels (both lungs are obstructed) at: (**a**) various sections of the lungs (including the particles that pass through the outlets) and (**b**) the first five generations. Particle diameter is 10 μm and density is 1000 Kg/m^3^

As a next step and as presented in Fig. 13(a), a number of models have been created with obstructions after the fifth, forth, third, second and first generation of airways which have been compared with the case of unobstructed airways. As observed, the studied model seems to have an asymmetric distribution of particles in all cases with slightly less particles deposited on the left lung. This observation further underlines the results of the analysis of the previous paragraph since the introduction of obstructions in the right lung completely counteracts the inherent asymmetry of the initial model. Furthermore, and as indicated in Fig. 13(b), a maximum deposition on the 5th generation is accomplished, when obstructions occur all over the pulmonary system. It is important to mention that the deposition of particles on the 5th generation of the obstructed lungs is minimum (5.37% of total) when obstructions occur only in the 5th generation followed by the case where obstructions occur in all the central airways modelled (1st up to 5th generations of airways).

#### Variations of particles diameter and density

**Fig. 14:**
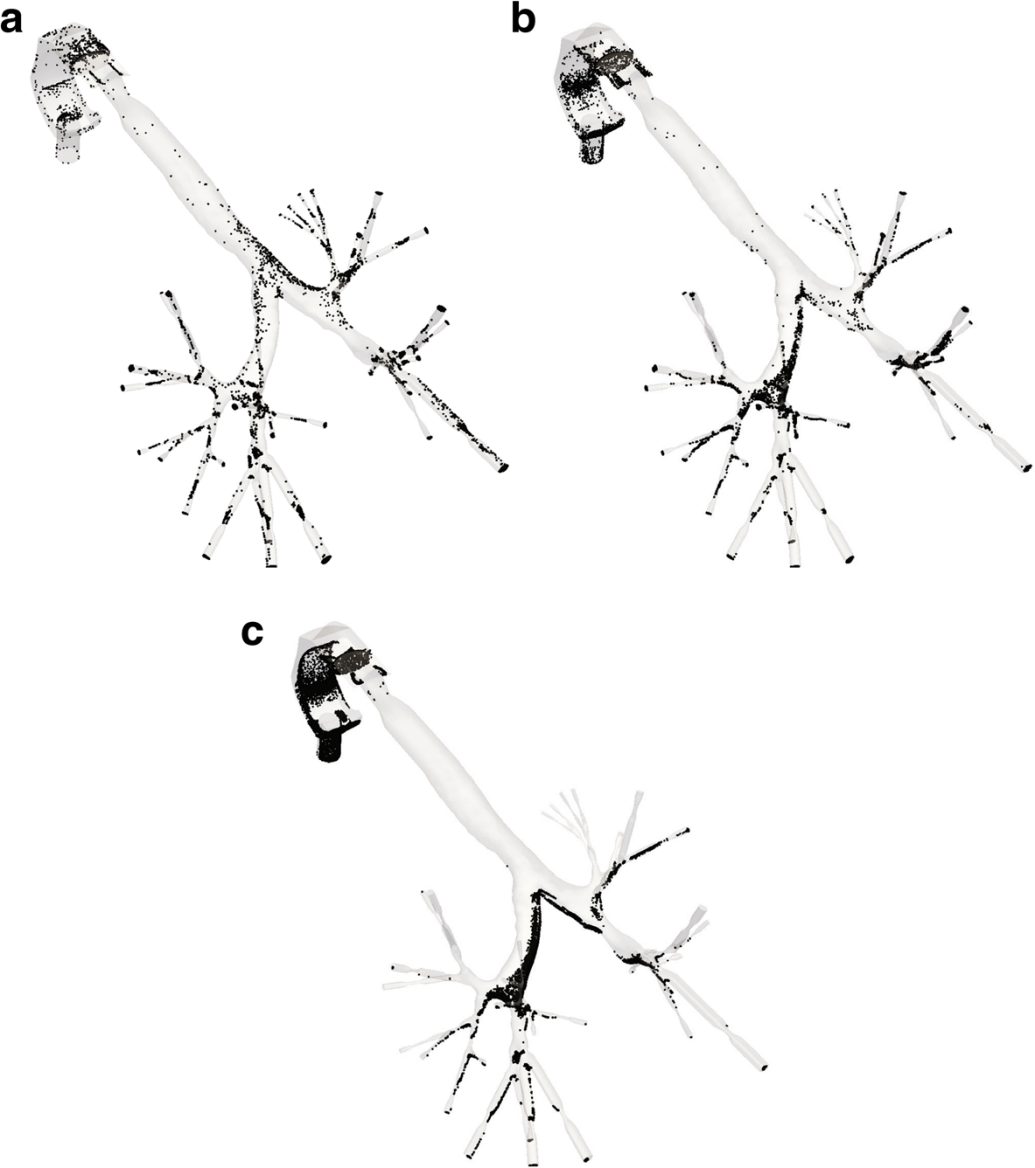
Particles position after the end of the inhalation period for diverse values of particles diameter, when obstructions in both lungs occur after the 2nd generation and the air flowrate is 6.06 L/min: (**a**) *d* = 1 μm, (**b**) *d* = 10 μm and (**c**) *d* = 20 μm

A parametric analysis of the particles characteristics is performed by means of several simulations for each lung model examined in the previous paragraph, where a 10 μm particles diameter was considered. In this context, the impact of the particles diameter on particle DF is assessed in conjunction with the effects of the particle density variations. For each case, particle DF is acquired for several generations as well as for distinct lung sections. Figure 14 illustrates particles depositions for three distinct values of diameter: *d* = 1 μm, *d* = 10 μm and *d* = 20 μm. These results make evident the importance of particle sizes on their distribution between the upper and lower airways, showing a great increase of DF on the upper airways when the size of the particle is increased.

**Fig. 15:**
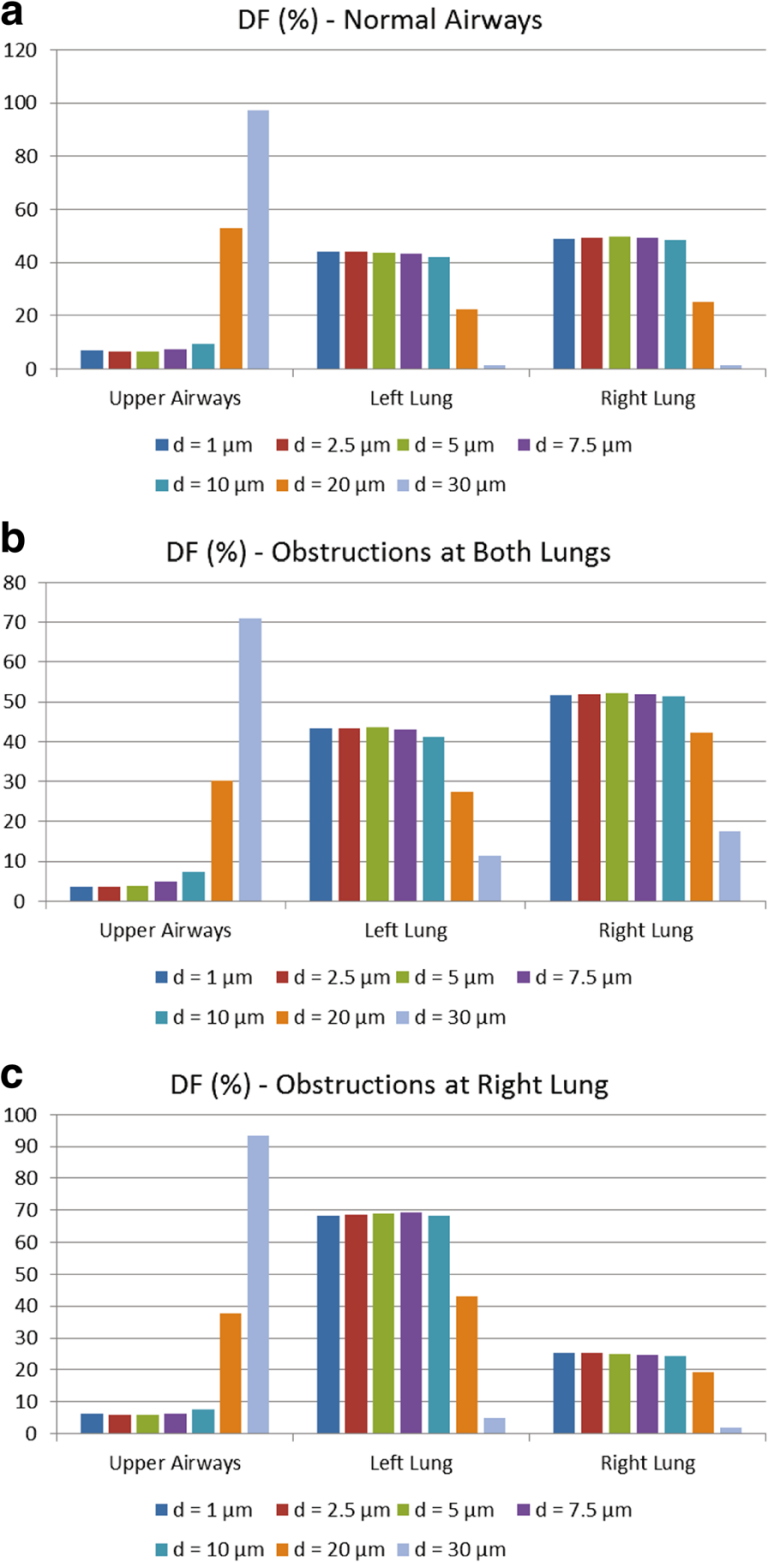
Particles size effects on deposition fraction over distinct lung sections (including the particles that pass through the outlets): (**a**) Normal airways, (**b**) narrowed airways (both lungs are obstructed) and (**c**) partially narrowed airways (right lung is obstructed only)

More specifically, Fig. 15 illustrates the DF for a range of particle sizes and for the instances of the normal lung model, the model with obstructions in both lungs and the model with obstructions only in the right lung. These observations denote that the majority of the particles with diameters from 1 μm to 10 μm are capable of passing through the upper airways, and they are distributed over the generations of the central airways. On the other hand, particles with diameters between 20 μm and 30 μm are mostly retained on the upper airways. Therefore, medical researchers could utilize these results to identify the region of maximum deposition, along with the proper diameter of the medication particles and form new and innovative treatment approaches that take into account the personalized characteristics of patient lungs.

**Fig. 16:**
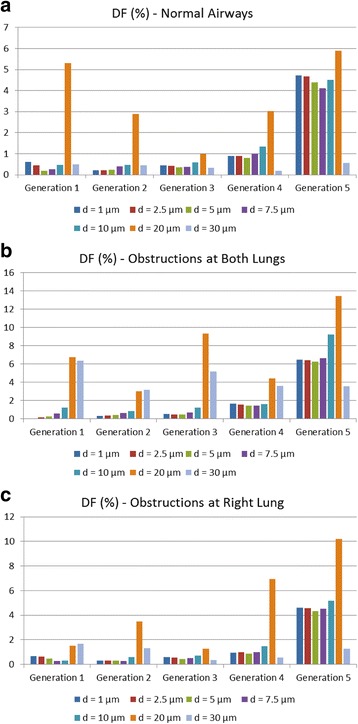
Particles size effects on deposition fraction over the lung’s generations for distinct cases: (**a**) Normal airways, (**b**) narrowed airways (both lungs are obstructed) and (**c**) partially narrowed airways (right lung is obstructed only)

By inspecting the diagrams of Fig. 16, the distribution of particles within the lower airways is analyzed across the first five generations. A maximum deposition is attained on every generation and for all cases examined herein, when *d* = 20 μm. This notice should be considered in combination with the fact that the majority of particles in this case are already deposited before they reach the lower airways, as illustrated in Fig. 15. Also, it is deduced that particles with diameters from 1 μm to 10 μm, exhibit an almost identical behavior when deposited on the first generations. However, particles with diameters from 20 μm to 30 μm, present a different deposition pattern, since most particles are gathered on the upper airways. Among the generations examined, the 5th one shows a maximum deposition of the substance. In general, when *d* increases, DF is enhanced even though it shows a local minimum.

**Fig. 17:**
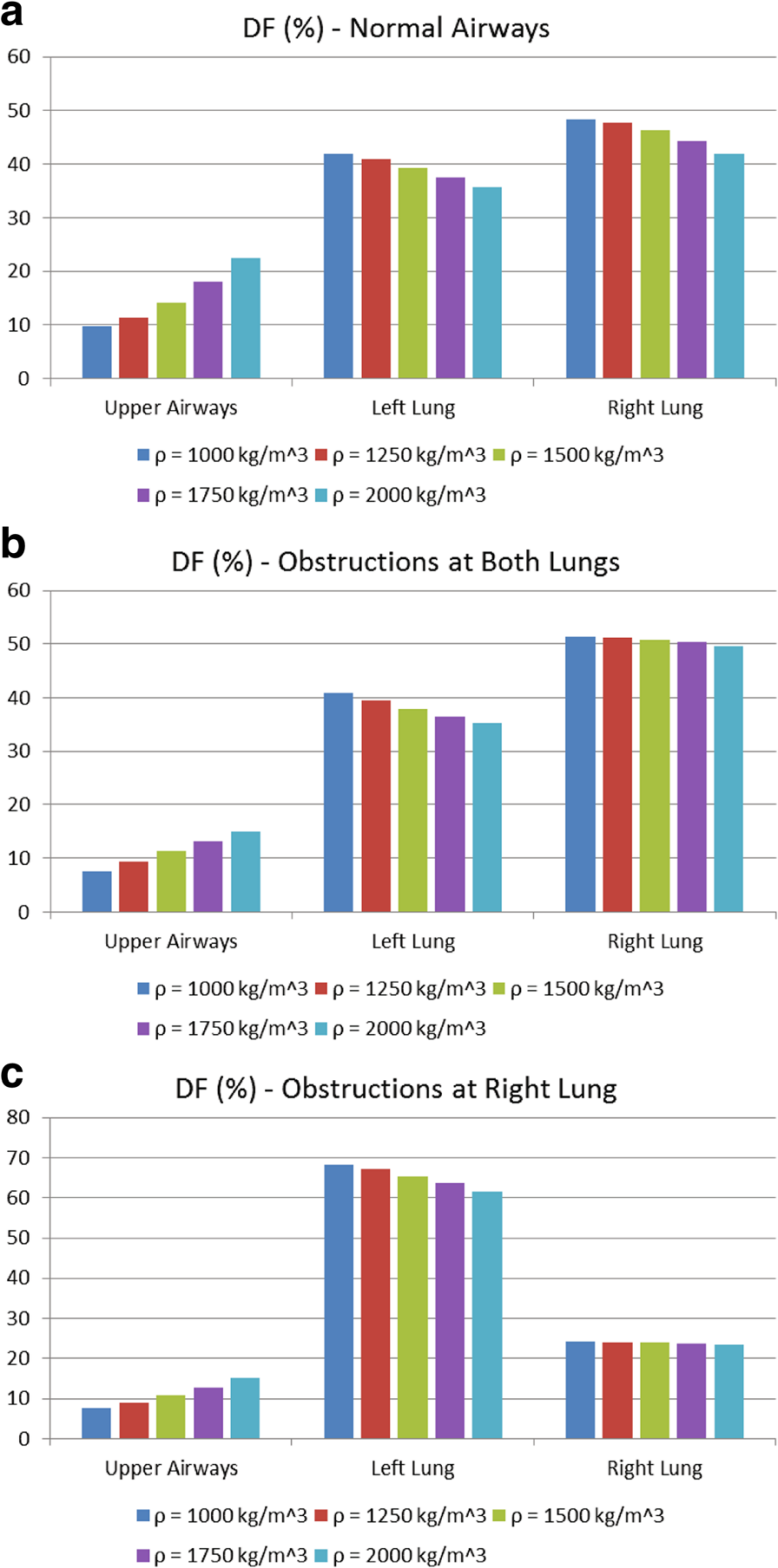
Particles density effects on deposition fraction over distinct lung sections (including the particles that pass through the outlets): (**a**) Normal airways, (**b**) narrowed airways (both lungs are obstructed) and (**c**) partially narrowed airways (right lung is obstructed only)

**Fig. 18:**
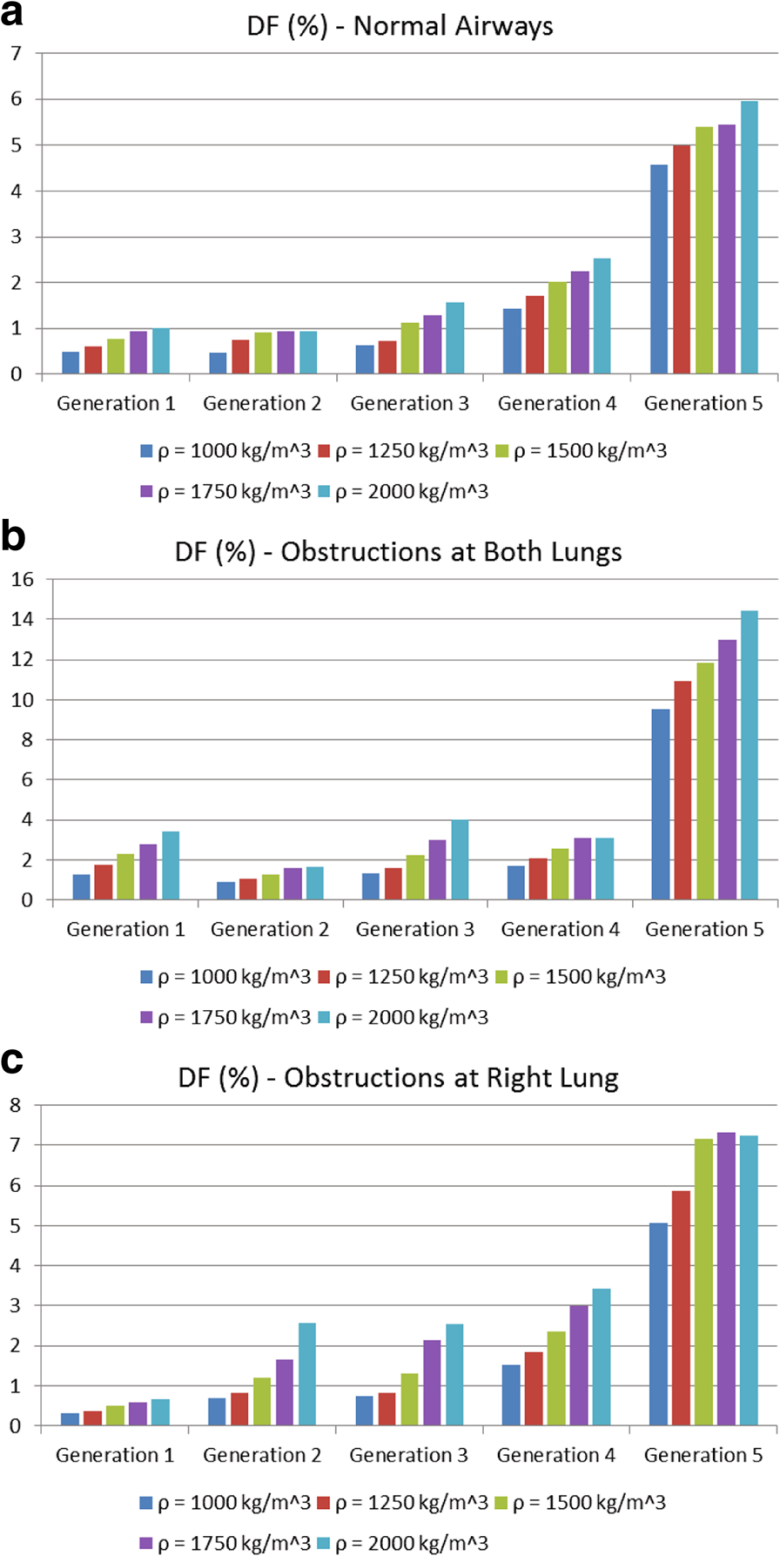
Particles density effects on deposition fraction over the lung’s generations for distinct cases: (**a**) Normal airways, (**b**) narrowed airways (both lungs are obstructed) and (**c**) partially narrowed airways (right lung is obstructed only)

Finally, an additional analysis is performed regarding the variation of the particles density between 1000 Kg/m^3^ to 2000 Kg/m^3^, as depicted in Figs. 17 and 18. More specifically, DF on the upper airways exhibits higher values, when *ρ* is increased, as depicted in Fig. 17. From the results presented in Fig. 18, it is deduced that DF is enhanced on every generation and for all cases examined herein, when *ρ* is augmented. These results can be used as an initial step for future detailed analysis by medical researchers for the in depth understanding of medication effectiveness in regards to the particle densities.

## Discussion

In the present study several detailed models of the airways obstructions associated with pulmonary diseases have been developed utilizing a custom geometry processing sequence, whereas the breathing process of airborne particles has been assessed numerically via CFD and FPT algorithms. Apart from the evaluation of the airflow behavior, the study focused on the properties of inhaled particles as an effective assessment tool for the optimization of treatment with the appropriate substances and densities. Two obstructed groups of models were examined, i.e. models with obstructions in both lungs and a model with obstructions in the right lung only. Moreover, a detailed parametric analysis identified the effects of the particles size and density on the substances deposition in the upper airways as well as the five first generations of the lower airways.

The results of the study confirmed the effects of obstructions in breathing capability of patients, as well as the discrepancy between the left and right lung in terms of airflow and particles deposition. Several DF assessments clarified that most particles are guided on the one lung, when narrowings are detected on the other side only and thus, any medication is shown to be directed away from the problematic blocked area. These results form the basis for further clinical investigations in regards to the personalized effectiveness of inhaled medication associated to specific lung characteristics of patients. The vision of the current work is to form the basis for a clinical methodology that will allow the in depth analysis of personalized lung models, so that medical personnel could select and provide appropriate medication (particle size and density) by quantifying the particle deposition capability on different parts of the patient’s lungs. This feature envisages a very important and innovative aspect to the personalization treatment of lung diseases by maximizing the efficiency of the inhaled medication on a regional perspective.

Even though the derived lung models provide information about the first five generations, adequate deductions have been drawn that could be extended in the future with more thorough analysis of more detailed lung models including higher airway generations. Such an extension of the models to higher generations of the lungs may provide further details and accuracy and could also reveal medication deposition phenomena that are inaccessible in the current investigation. In this context, an extended study for a large number of patients may allow the necessary quantification of inhaled medication into the lungs, which is required for extended personalized treatment.

Apart from the extension of the simulation scenarios to a wider spectrum of models and obstruction cases, future directions of the current study will include the investigation of outcomes in the clinical setting and with the involvement of patients. This approach aspires to verify the effects that the lung geometry may have on the actual effectiveness of inhaled medication and how the current results can be translated into useful and clinically significant methodologies for the optimization of management of respiratory diseases.

## Conclusions

The utilization of contraction algorithms to clarify the diverse conditions of lung obstructions has been presented in this paper. In addition, an effective assessment of the consequences of inhaled particles in a regional oriented perspective has been performed by accurate CFD and FPT simulations. For the case of obstructions in both lungs most particles are driven upon the narrow regions when symmetrical narrowings appear. Moreover, partial medication transport is accomplished when only one lung is obstructed showing a decreased deposition of particles in the areas of inflammation. An additional parametric analysis regarding the variation of particles size and density allowed the better interpretation of particle deposition within the pulmonary system. It has been shown that the specific characteristics of particles (particle size and density) significantly affect the point of the respiratory system that they are deposited. More specifically, larger particles of higher density are shown to be affecting mainly the upper airways, whereas substances of lower particle size and density are deposited mainly on the higher generations of the airways. These observations could be valuable when treating lung maladies with inhaled medical substances and are aiming to form the basis for the clinical investigation and testing of innovative personalized treatment approaches that take into account the size and density of particles of inhaled medications.

## Abbreviations

CFD: Computational fluid dynamics
COPD: Chronic obstructive pulmonary disease
DF: Deposition fraction
DNS: Direct numerical simulation
FPT: Fluid particle tracing
FVM: Finite volume method
HPC: High performance computer
LES: Large eddy simulation
MDCT: Multidetector computer tomography
RANS: Reynolds-averaged Navier-Stokes
RAS: Reynolds-averaged simulation
SDF: Shape diameter function
